# Enteroviral and herpes simplex virus central nervous system infections in infants < 90 days old: a Paediatric Investigators’ Collaborative Network on Infections in Canada (PICNIC) study

**DOI:** 10.1186/s12887-020-02151-4

**Published:** 2020-05-26

**Authors:** Dara Petel, Michelle Barton, Christian Renaud, Lynda Ouchenir, Jason Brophy, Jennifer Bowes, Sarah Khan, Ari Bitnun, Jane McDonald, Andrée-Anne Boisvert, Joseph Ting, Ashley Roberts, Joan L. Robinson

**Affiliations:** 1 Department of Pediatrics, Western University, London, Ontario Canada; 2 Department of Pediatrics, University of Montreal, Montreal, Quebec Canada; 3 Department of Pediatrics, University of Ottawa, Ottawa, Ontario Canada; 4 Children’s Hospital of Eastern Ontario Research Institute, University of Ottawa, Ottawa, Ontario Canada; 5 Department of Pediatrics, McMaster University, Hamilton, Ontario Canada; 6 Department of Pediatrics, University of Toronto, Toronto, Ontario Canada; 7 Department of Pediatrics, McGill University, Montreal, Quebec Canada; 8 Department of Pediatrics, University of British Columbia, Vancouver, British Columbia Canada; 9 Department of Pediatrics, University of Alberta, 4-590 ECHA, 11405-87 Ave, Edmonton, AB T6G 1C9 Canada

**Keywords:** Meningoencephalitis, Central nervous system infection, Meningitis, Neonate

## Abstract

**Background:**

The relative contribution of viruses to central nervous system (CNS) infections in young infants is not clear. For viral CNS infections, there are limited data on features that suggest HSV etiology or on predictors of unfavorable outcome.

**Methods:**

In this cross-sectional retrospective study, seven centers from the Pediatric Investigators Collaborative Network on Infections in Canada identified infants < 90 days of age with CNS infection proven to be due to enterovirus (EV) or herpes simplex virus (HSV) January 1, 2013 through December 31, 2014.

**Results:**

Of 174 CNS infections with a proven etiology, EV accounted for 103 (59%) and HSV for 7 (4%). All HSV cases and 41 (40%) EV cases presented before 21 days of age. Four HSV cases (57%) and 5 EV cases (5%) had seizures. Three (43%) HSV and 23 (23%) EV cases lacked cerebrospinal fluid (CSF) pleocytosis. HSV cases were more likely to require ICU admission (*p* = 0.010), present with seizures (*p* = 0.031) and have extra-CNS disease (*p* < 0.001). Unfavorable outcome occurred in 12 cases (11% of all EV and HSV infections) but was more likely following HSV than EV infection (4 (57%) versus 8 (8%); *p* = 0.002).

**Conclusions:**

Viruses accounted for approximately two-thirds of proven CNS infections in the first 90 days of life. Empiric therapy for HSV should be considered in suspected CNS infections in the first 21 days even in the absence of CSF pleocytosis unless CSF parameters are suggestive of bacterial meningitis. Neurodevelopmental follow-up should be considered in infants whose course of illness is complicated by seizures.

## Background

The prevention of bacterial meningitis by conjugate vaccines has resulted in viruses accounting for an increasing proportion of central nervous system (CNS) disease in childhood [1]. Improvement in viral diagnostics has made this trend more apparent. Previous studies of viral CNS disease were limited by small sample size, included cases where the etiology was not proven or did not focus on infants [2, 3]. The most common viruses associated with CNS infections are enteroviruses (EV), which most frequently manifest as self-limited aseptic meningitis with no recognized long-term sequelae. By contrast, herpes simplex virus (HSV) CNS infections result in significant morbidity and mortality, especially if acyclovir therapy is delayed [4]. It is therefore vital that clinicians know what clinical and laboratory features should prompt them to start empiric acyclovir.

This was a cross-sectional analysis to identify infants less than 90 days of age with proven CNS infections. This age range was selected as diagnosis of CNS infections is particularly challenging in young infants. We sought to a) determine the relative contribution of HSV and EV to microbiologically-confirmed CNS infections, b) provide a comparative analysis of the epidemiology and outcome of HSV and EV CNS infection, c) describe factors associated with HSV aetiology and d) identify factors associated with unfavorable outcome.

## Methods

### Study population and design

Seven paediatric academic centres within the Paediatric Investigators Collaborative Network on Infections in Canada (PICNIC) retrospectively enrolled hospitalized infants < 90 days of age with microbiologically-confirmed CNS infection January 1, 2013 through December 31, 2014. Cases were identified using appropriate discharge diagnostic codes from the International Statistical Classification of Diseases and Related Health Problems, Tenth Revision (ICD 10) (Appendix A) and charts were then reviewed. A previous publication described cases of bacterial CNS infection as proven if bacteria were detected from cerebrospinal fluid (CSF) or brain abscess by means of culture or PCR or probable if CSF pleocytosis was present, along with bacterial growth from another sterile site [5]. For the purposes of this study, we included all proven cases of HSV or EV CNS infection based on the identification of a virus in the CSF by polymerase chain reaction (PCR) during life or in the brain tissue using PCR at autopsy. All study centres offered routine PCR testing for HSV and EV. None used multiplex PCR. Only two centres offered HPeV testing during the study period so it was not possible to compare HPeV cases to other viral cases. Cases with coinfection were excluded unless the investigator deemed that a virus was the main pathogen. There were no other exclusion criteria.

Ethics board approval was obtained from all participating centres with the primary approval coming from the Health Research Ethics Board of the University of Alberta (Study number PRO00055909).

### Study definitions

1. Case classification: a) early onset if diagnosis was made within the first 6 days of life, b) late onset if diagnosis was made day 7 through 29 of life and c) very late onset if diagnosis was made day 30 through 90 of life.
2. Infants were considered to have extra-CNS disease if there was microbiological, clinical or other laboratory findings consistent with viral disease at other sites.
3. Infants who had i) seizures or ii) head imaging suggesting parenchymal involvement were presumed to have meningoencephalitis. All other cases were deemed to have meningitis.
4. Unfavourable outcomes were defined as:
  Neurodevelopmental sequelae (any one of hearing loss, visual impairment, other neurological sequelae such as extensive intracranial haemorrhage or hydrocephalus, or developmental delay noted at follow-up) OR
  Death

### Data collection and analysis

Demographic, clinical, microbiological, head imaging reports, treatment, outcome and any available follow-up data were extracted from medical records and entered into Research Electronic Data Capture (REDCap) by each participating center. Follow-up data were collected at variable time points depending upon local protocols and parental compliance with follow-up. Follow-up data were not available if the neonatal follow-up program was not in the institution where the infant was admitted.

Two separate although related comparative analyses were undertaken comparing clinical features and outcome by etiology (HSV versus EV). Descriptive analysis was conducted. Chi-square or Fisher’s exact test was used to compare categorical variables and non-parametric tests were used to compare continuous variables (Mann–Whitney U test). Exploratory analysis was conducted using univariate analyses and where sample size allowed, multivariate analyses to identify clinical, laboratory or outcome differences between EV and HSV cases were conducted using factors identified as significant in univariate analysis. Additionally, we used univariate analysis to explore potential factors associated with an unfavorable outcome overall. We adjusted for multiple comparisons using Bonferroni correction. Epi-info version 7 (Centers for Disease Control and Prevention) was used for statistical analysis.

## Results

### Relative contribution of viruses to microbiologically-confirmed CNS infections

There were 174 cases of proven CNS infections in infants < 90 days old, of which 111 (64%) were viral in origin. One case was excluded due to coinfection with group B streptococcus and EV. The most common identified viral pathogen was EV (*N* = 103; 93%) followed by HSV (*N* = 7; 6%) and human parechovirus (HPeV) (*N* = 1; 1%). The HSV cases included 3 with HSV1 (1 with isolated CNS disease, and 2 with disseminated disease) and 4 with HSV2 (1 with isolated CNS disease, and 3 with disseminated disease).

### Descriptive analysis of EV and HSV CNS infections

#### Demographics

**Fig. 1:**
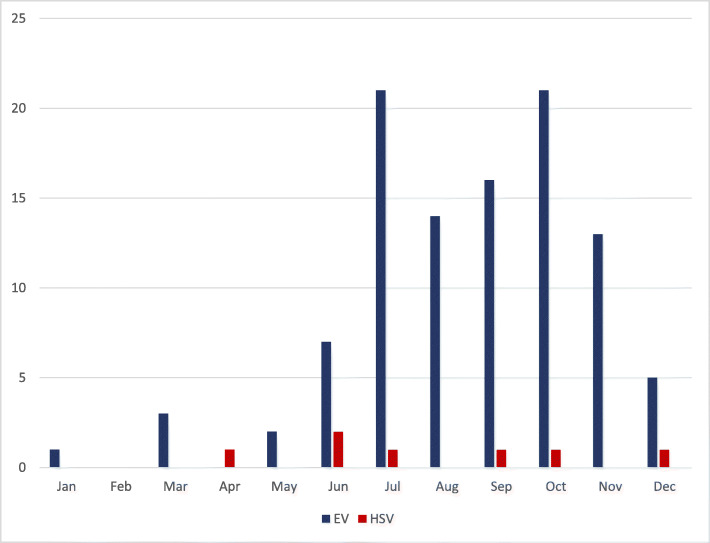
Seasonality of HSV and enteroviral CNS infections in infants < 90 days of age

The median birth weight was 3343 g (range 1670-4900 g) and median gestational age was 37 weeks (range 29–40 weeks). Sixteen infants were preterm (15%). Infants presented at a median age of 22.5 days (range 3–84 days), with 5 cases occurring during the birth hospitalization (all were EV infection on day 3 to day 21 of life in infants born at 31 to 35 weeks GA). HSV cases presented earlier than EV cases (median 14 days versus 25 days of life; *p* = 0.02) (Table 1). Fifty-two (50%) of EV cases and 3(43%) of HSV cases presented August through October (Fig. 1).

**Table 1:** Comparison of demographic, clinical and outcome features in infants with HSV and EV meningitis by univariate analysis

	Characteristics	HSV *N* = 7	EV *N* = 103	*P*-Value^a^
Demographic	Male gender, n (%)	3 (43)	54 (52)	0.71
	Age at onset (d), median (IQR)	14 (6–19)	25 (12–33)	0.02
	Overall	16 (6–19)	10 (6–27)	1.0
	Subset fulfilling meningoencephalitis criteria [Proportion (%) age < 28d]	[6/6 (100)]	[6/7 (86)]	1.0
	Gestation (wks), median (IQR)	37 (37–38)	37 (29–38)	0.31
Clinical	ICU admission, n (%)	4 (57)	12/98 (12)	0.010
	Seizures, n (%)			
	Had at least one seizure at any time	4 (57)	5 (5)	.001
	Had a seizure in first 72 h of admission	2 (29)	3 (3)	0.03
	Had at least one seizure during the admission	3 (43)	5 (5)	0.008
	Had seizures only after discharge	1 (16)	0 (0)	0.06
	Multisystemic infection^b^	5 (71)	8 (8)	< 0.001
	Coinfection with bacteria or fungus, n (%)	1 (14)	4 (4)	0.28
Imaging	Abnormal head imaging^c^, n (%)	4/7 (57)	5/26 (19)	0.068
	Meningoencephalitis**,** n (%)	6/7 (86)	7 (7)	< 0.001
Hospital stay	Length of stay (d), median (IQR)	25 (21–43)	3 (3–5)	< 0.001
Follow-up	Follow-up (mo), median (IQR)	16 (10–24)	6 (1–12)	0.03
Neurodevelopmental or neurological sequelae	Neurodevelopmental abnormalities at discharge or follow-up^d^, n (%)	3/6 (50)	7/102 (7)	0.01
Outcomes	Death or neurological complications^e^ or neurodevelopmental abnormalities, n (%)			
	All infants	4/7 (57)	8 (8)	0.002
	Infants with encephalitis	4/6 (67)	4/7 (57)	1.00

Legend: *CSF* cerebrospinal fluid, *EV* enterovirus, *HSV* herpes simplex virus, *IQR* interquartile range, *mo* months

^a^For comparison of proportions, Fishers exact test (2-sided) was used; for comparison of medians, Mann-Whitney test was used

^b^These were identified as independent risk factors after controlling for age and ICU admission, respectively

^c^This comparison was limited to those abnormalities that were consistent with CNS infection

^d^All infants with long-term seizures had neurodevelopmental delay (range mild to profound)

^e^ After adjusting for multiple comparisons (Bonferroni correction), these variables remained significant

#### Maternal history

Among HSV cases, three (38%) were born to mothers with active genital lesions documented at or within 7 days of delivery (Table 1). Data on the reasons for the mode of delivery were not collected. One of the three mothers had recurrent HSV1 disease and was not compliant with acyclovir prophylaxis; her infant presented with HSV1 on day 4 of life after vaginal delivery. The other two mothers had first clinical episode of genital HSV within 7 days of delivery. Their infants received no screening or empiric treatment and presented with HSV2 on days 6 and 9 after caesarean and vaginal delivery, respectively; the duration of rupture of membranes was not available. For EV infection, 3 mothers had documented illness compatible with EV within 10 days prior to delivery; their infants presented on days 5, 6 and 8.

#### Timing of presentation

Early-onset infection occurred in 10 of the 110 infants (8 EV and 2 HSV). The EV cases all presented after day 2 of life and 3 of the 8 had severe disease including: fatal myocarditis, shock with coagulopathy and meningoencephalitis. The two early-onset cases with HSV infection presented on days 4 and 6.

For late-onset disease, there were 63 cases of EV meningitis and 5 cases of HSV meningoencephalitis. All infants (*N* = 32) with very late onset infection had EV. All HSV cases presented before 21 days of age.

#### Clinical features

There were 9 (8%) cases with seizures. Eight had seizures during the admission for the CNS infection (5 with EV and 3 with HSV) and the ninth developed seizures after discharge coinciding with CNS HSV relapse. For the 4 HSV cases, 2 had seizures only within the first 72 h following diagnosis, 1 after 72 h but prior to hospital discharge and as mentioned previously, one case only after discharge. Five of 103 infants with EV had seizures (5%), with 3 presenting in the first 72 h following diagnosis and 2 presenting after 72 h but prior to hospital discharge. The age at diagnosis of CNS infection for these 5 cases was 5, 10, 10, 14 and 84 days.

There were 14 (12%) infants with extra-CNS involvement. Five infants had extra-CNS HSV infection, consisting of vesicular lesions without other extra-CNS involvement (*N* = 1), transaminitis and pneumonitis (*N* = 1), transaminitis and vesicles (*N* = 1) and transaminitis, pneumonitis and coagulopathy (*N* = 2). Coagulopathy was complicated by spontaneous intracranial haemorrhages (intraventricular and parenchymal) in one of these two infants. Extra-CNS manifestations in EV cases included rash (*N* = 2), pneumonia (*N* = 2), shock with coagulopathy (*N* = 2), myocarditis (*N* = 2) and transaminitis (*N* = 1). The median age of onset of the 7 cases with organ involvement (omitting the 2 with skin involvement) was 9 days (range 5–73 days). Extra-CNS involvement was more likely in HSV than EV cases (*p* < 0001), even if skin involvement was not considered (4 (57% versus 5 (6%); *p* = 0.001) (Table 1).

#### Microbiology

All cases were diagnosed using PCR analysis of CSF. HSV PCR testing was also positive on skin lesions in two infants and from the conjunctiva of one (in the absence of ophthalmological abnormalities). EV typing was not available. Suspected urinary tract coinfections occurred in 4 infants with EV infection (Table 1). Systemic candidiasis complicated the course of one infant with HSV meningoencephalitis with liver failure, coagulopathy and intraventricular haemorrhages requiring external ventricular drain (EVD) placement. *Candida albicans* was isolated from blood and CSF obtained from EVD just prior to demise. Newborn screen, immunoglobulin assay and flow cytometry failed to identify an underlying immunodeficiency in this fatal case.

#### CSF findings

The median values for cell count, glucose and protein on the initial CSF were not significantly different between HSV and EV (Table 2). Thirty-six (33%) infants (4 with HSV and 32 with EV) had CSF white blood cell (WBC) counts less than 30 × 10^6^/L. Notably, 5 (5%) of infants with EV infections had CSF WBC > 2000 X 10^6^/L.

**Table 2:** Comparison of initial cerebrospinal fluid findings in infants with HSV and EV central nervous system infections by univariate analysis

Parameter	Cerebrospinal (CSF) Findings	HSV *N* = 7	EV *N* = 100^a^	*P*-Value^b^
CSF white blood cell count	CSF white blood cell (WBC) (× 10^6^/L) at diagnosis, median (IQR)	26 (2–146)	153 (17.5–422)	0.08
	CSF WBC, n (%)			
	> 9 × 10^6^/L	4 (57)	79 (79)	0.18
	> 15 × 10^6^/L	4 (57)	76 (76)	0.37
	> 100 × 10^6^/L	3 (43)	57 (57)	0.70
	> 500 × 10^6^/L	0	22 (22)	0.34
	> 1000 × 10^6^/L^c^	0	10 (10)	1.00
	> 2000 × 10^6^/L^d^	0	6 (6)	1.00
	Median percentage polymorphonuclear contribution to CSF cell count	6 (3–14)	25 (6–51)	0.04
	CSF pleocytosis^e^	4 (57)	77 (77)	0.36
	CSF cell count with polymorphonuclear dominance (> 50%), n (%)	0/7 (0)	23/86 (27)	0.19
CSF red blood cell count	CSF red blood cell count (×10^6^/L), median (IQR)	26 (1–6950)	23 (2–138)	0.66
CSF glucose	CSF glucose (mmol/L), median (IQR)	2.4 (2.1–2.8)	2.4 (2.2–2.7)	0.90
	CSF glucose < 2.1 mmol/L^c^, n (%)	0/7 (0)	10 (10)	0.91
CSF protein	CSF protein (g/L), median (IQR)	0.76 (0.53–0.90)	0.79 (0.59–1.02)	0.86
	Protein > 1 g/L^c^, n (%)	1 (14)	25/98 (25)	0.68

Legend: *CSF* cerebrospinal fluid, *HSV* herpes simplex virus, *IQR* interquartile range, *WBC* white blood cell count

^a^Three of the EV cases had CSF sent only for microbiological analysis; so only 100 cases had CSF analysis that included a cell count, protein or glucose level; 2/7 (29%) of the EV cases that were classified as meningoencephalitis had CSF WBC < 30 × 10^6^/L

^b^For comparison of proportions, Fishers exact test (2-sided) was used; for comparison of medians, Mann-Whitney test was used

^c^ EV cases were more likely than HSV cases to have one or more of the parameters (cell count > 1000 × 10^6^/L, Glucose < 2.0 mmol/L and CSF Protein > 1.0 g/L. ≥1) that suggested bacterial meningitis (65 (64%) versus 1 (13%); *p* = 0.006)

^d^ EV cases with CSF WBC > 2000 × 10^6^/L had median CSF WBC of 2630 (range 2020–6400) × 10^6^/L

^e^ CSF pleocytosis was defined as CSF white cell count > 15 × 10^6^/L for infants 0–28 days of age and > 9 × 10^6^/L for infants beyond neonatal period. ^6^ Of 74 neonates with spinal taps, 22 (30%) neonates had CSF WBC < 15 × 10^6^/L and 4/33 (12%) infants >28d old had CSF WBC < 9 × 10^6^/L

#### Head imaging

Thirty-three (30%) of the 111 infants had head imaging performed (HSV (*N* = 7) and EV (*N* = 26)). Among the cases of HSV, magnetic resonance imaging was abnormal in 4/6 (67%) and appeared consistent with infection; the seventh case had only a head ultrasound which was normal. Among the cases of EV, 7/26 (27%) had abnormalities detected on imaging but only 5 (19%) of these were attributed to infection (diffusion restriction abnormalities).

#### Meningoencephalitis

Thirteen infants (HSV = 6; EV = 7) fulfilled the study criteria for meningoencephalitis. The EV cases presented at a median of 10 days of age (range 5 to 84 days). One case of disseminated HSV2 infection did not meet our definition of meningoencephalitis as the infant did not have documented seizures and only had a normal head ultrasound documented, but did not have MRI or CT imaging performed. Infants with meningoencephalitis were younger (*p* = 0.012), more likely to require ICU admission (*p* < 0.001), more likely to have disseminated disease (*p* = 0.007) and more likely to die or have developmental delay (8 (62%) vs 4 (4%); *P* < 0.001) than those without meningoencephalitis. Poor long term outcome in survivors with meningoencephalitis was equally likely whether the cause was HSV (3/5; 60%) or EV (4/7; 57%) (*p* = 1.0). Adjusting for multiple comparisons, these associations remained significant.

#### Antiviral treatment and prophylaxis

All HSV cases received acyclovir treatment for a median of 21 days (range 21–51 days). One infant received acyclovir until demise on day 42 of acyclovir therapy. Acyclovir resistance was first tested for on a sample just prior to death and was proven to be present. Another infant did not have documented CSF clearance until 51 days of therapy. For the other 5 cases, repeat testing done between 19 and 22 days of treatment confirmed successful clearance of HSV from CSF. Three (50%) surviving infants were documented to have been discharged on oral acyclovir as prophylaxis for minimum 6 months.

#### Outcome

There were 2 deaths (2%), one from disseminated EV (a 6-day old infant with myocarditis who required extracorporeal membrane oxygenation) and one from HSV2 (the infant with systemic candidiasis and with persistent HSV detection in CSF until death at day 48 of life). Autopsies were not performed. Virologically-proven recurrence of HSV1 meningoencephalitis presenting as infantile spasms occurred in 1 (33%) of the 3 infants who received oral acyclovir until 6 months of life; this occurred 2 weeks after oral acyclovir was discontinued. Ten (9%) of the 108 surviving infants had neurodevelopmental sequelae documented at discharge or follow-up (Table 1). Neurodevelopmental outcomes were not available for infants who had HSV persistence documented in CSF as the single survivor was lost to follow-up. All 3 of the HSV (2 HSV2; 1 HSV) and 1 of the EV survivors with neurodevelopmental sequelae developed seizure disorders requiring anticonvulsant therapy. Overall, unfavorable outcome occurred in 12 cases (11% of all EV and HSV infections) but was more likely following HSV than EV infection (4 (57%) versus 8 (8%); *p* = 0.002) (Table 1). Three (75%) of four HSV cases with unfavorable outcome were caused by HSV2. One of 3 (33%) cases of HSV1 had poor outcome compared to 3 of 4 (75%) cases with HSV2. All cases of EV meningoencephalitis survived. There were no differences by pathogen in the incidence of poor neurodevelopmental outcomes in surviving infants with presumed encephalitis (3/5 (60%) HSV vs 4/7 (57%) EV; *p* = 1.0). Eight (62%) of 13 infants (HSV = 4; EV = 4) with meningoencephalitis had unfavorable outcome.

#### Factors associated with HSV aetiology

In univariate analysis, HSV cases were more likely than EV cases to require intensive care unit (ICU) admission (*p* = 0.010), have seizures at any time (*p* = 0.001), have extra-CNS disease (*p* < 0.001) and have unfavorable outcome (*p* < 0.001) (Table 1). The latter three remained significant after correcting for multiple comparisons. Seizures (*p* = 0.005) and extra-CNS disease (*p* = 0.002) remained significant after controlling for ICU admission.

Among infants < 30 days of age (*N* = 78), the presence of seizures or extra-CNS disease was more likely in HSV than in EV CNS infection (6 of 7; (86%) versus 10 of 71; 14%); *p* < 0.001).

### Factors associated with unfavorable outcome

In the univariate analysis, several factors were identified (Table 3). After adjusting for multiple comparisons, the factors associated with unfavorable outcome included younger age (*p* = 0.003), HSV etiology (*p* = 0.002), seizures (*p* < 0.001), ICU admission (*p* < 0.001) and meningoencephalitis (*p* < 0.001) (Table 3). The latter 3 remained significant when analysis was limited to the subgroup of infants with EV CNS infections (Table 4 – The sample size limited multivariate analysis).

**Table 3:** Demographic, clinical and laboratory factors associated with unfavorable outcome following viral CNS infection by univariate analysis

Factors	Unfavorable Outcome *N* = 12	Favorable outcome *N* = 98	*P*-Value^a^
Age at onset (d), median (IQR)	9 (5.5–18.5)	25 (14–33)	0.003*
History of seizure at onset or during treatment, n (%)	7/12 (58)	2/98 (2)	< 0.001*
Abnormal imaging, n (%)	7/11 (64)	4/22 (18)	0.02
Meningoencephalitis, n (%)	8/12 (67)	6/98 (6)	< 0.001*
^a^Intensive care unit admission, n (%)	7/11 (64)	9/95 (9)	< 0.001*
Underlying virus, n (%)			
HSV	4/12 (33)	3/98 (3)	0.002*
HSV1	1/12 (8)	2/98 (2)	0.29
HSV2	3/12 (25)	1/98 (1)	0.004
EV	8/12(67)	95/98 (97)	0.002
CSF white blood cell count (× 10^6^/L)^b^, median (IQR)	104 (27.5–762)	147 (11–358)	0.75
CSF protein (g/L)^b^, median (IQR)	1.0 (0.64–1.27)	0.74 (0.56–0.95)	0.12
^b^CSF glucose (mmol/L), median (IQR)	2.1 (2.0–2.45)	2.4 (2.2–2.75)	0.03
Extra-central nervous system disease, n (%)	5/12 (42)	8/99 (8)	0.005

Legend: *CSF* cerebrospinal fluid, *HSV* herpes simplex virus

*These variables remained significant at a *p* value < 0.004 after Bonferroni correction applied for multiple comparisons

^a^For comparison of proportions, Fishers exact test (2-sided) was used; for comparison of medians, Mann-Whitney test was used

^b^The presence of one of more of parameters suggestive of bacterial meningitis (cell count > 1000 × 10^6^/L, Glucose < 2.0 mmol/L and CSF Protein > 1.0 g/L) in infants with EV or HSV infection were not associated with unfavorable outcome

**Table 4:** Demographic Clinical and Laboratory Factors Associated with Unfavorable Outcome Following Enteroviral CNS Infection

Factors	Unfavorable Outcome *N* = 8	Good outcome *N* = 95	*P*-Value^a^
Age at Onset (d), median (IQR)	9 (5.5–19.5)	25 (14–34)	0.02
History of seizure at onset or during treatment, n (%)	3/8 (38)	2/95 (2)	0.003*
Abnormal imaging, n (%)	3/7 (43)	4/19 (21)	0.34
Meningoencephalitis, n (%)	4 (50)	3 (95)	< 0.001*
Intensive care unit admission, n (%)	4/7 (57)	8/91 (9)	0.004*
CSF white blood cell count × 10^6^/L^b^, median (IQR)	180 (41–1271)	153 (15.5–393.5)	0.36
CSF protein (g/L), median (IQR)	1.10 (0.73–1.27)	0.76 (0.59–0.99)	0.13
CSF protein over 1 g/L^b^, n (%)	5/8 (63)	20/90 (22)	0.02
CSF glucose (mmol/L)^b^, median (IQR)	2.1 (1.95–2.29)	2.45 (2.20–2.75)	0.01
Extra-CNS disease, n (%)	2/8 (25)	6/95(6)	0.12

Legend: *CNS* central nervous system, *CSF* cerebrospinal fluid

*These variables remained significant at a *p* value of < 0.005 after Bonferroni correction applied for multiple comparisons

^a^For comparison of proportions, Fishers exact test (2-sided) was used; for comparison of medians, Mann-Whitney test was used

^b^ The presence of one of more of parameters suggestive of bacterial meningitis (cell count > 1000 × 10^6^/L, Glucose < 2.0 mmol/L and CSF Protein > 1.0 g/L) in infants with EV was not associated with unfavorable outcome (8 (100%) infants who had unfavourable outcome with EV fit this criteria versus 57 (62%) who had favorable outcome; *p* = 0.05)

## Discussion

Viral infections accounted for about two-thirds of CNS infections in the first 90 days of life where a CSF pathogen was detected in the current study. Trends in Canada are not clear but in a population-based United Kingdom (UK) study, the authors document a dramatic rise in admissions for viral meningitis in infants between 2005 and 2011 [6]. They show a major increase in the proportion of cases of viral meningitis recognized to be due to EV over time, from 90 (3%) of 2770 admissions for viral meningitis in 1968–1985 to 811 (47%) of 1716 viral meningitis admissions in 2007–2011 [6]. These changing trends probably reflect the UK adoption of molecular diagnostic screening for viral meningitis resulting in increased detection over conventional viral culture methods which were not consistently applied in earlier years [7]. Further, molecular testing has facilitated the detection of viruses like HPeV that are missed by viral isolation techniques [7].

Consistent with prior literature, CNS infection with HSV was much more likely than infection with EV to lead to meningoencephalitis and long-term neurodevelopmental morbidity or death [3, 8–11]. However, among the subgroup of EV cases with meningoencephalitis, outcomes were comparable to cases of HSV meningoencephalitis. Identifying clinical or laboratory markers that distinguish HSV from non-HSV viral infections is vital to ensure that empiric acyclovir is started at presentation in all HSV cases [4]. We identified younger age, seizures, ICU admission and the presence of extra-CNS features as factors associated with HSV infection; however, only seizures and extra-CNS disease remained significant in the multivariate analysis and 3 of 7 infants with HSV CNS disease (43%) did not have seizures. Most if not all HSV meningoencephalitis in the neonatal period comes from perinatal transmission, and in our study all presented by day 21 of life. There should be limited use of empiric acyclovir beyond the first month of life [12]. However, HSV meningoencephalitis can present at any age and in a 2018 study of 46 cases up to 60 days of age, the IQR was 9 to 24 days [13].

Most genital HSV infections are subclinical. A small percentage of neonatal HSV cases may arise from post-natal transmission from saliva [8]. Therefore, all infants should be assumed to be at risk of HSV infection irrespective of maternal history. In addition, as demonstrated in 4 of the 7 infants with CNS HSV in our cohort, the absence of CSF pleocytosis does not exclude CNS HSV infection. Furthermore, one case in our cohort had HSV detected by PCR on CSF analysis from day 5 of illness after a negative PCR on day 2 of illness. Thus, if the clinical picture is suggestive of HSV infection and initial CSF HSV testing returns negative, acyclovir should be continued until another CSF sample is retested to ensure that the original sample was not falsely negative [14]. The potential value of repeating CSF analysis towards the end of treatment course is exemplified by the two cases with persistent detection of HSV in CSF, although there are not studies to prove that continuing intravenous acyclovir beyond the usual 21-day course improve prognosis.

As noted in our fatal HSV2 case, the possibility of acyclovir resistance should be considered in children with persistent detection of the virus in CSF [15–19]. While rare, the possibility of acyclovir resistance needs to be kept in mind as alternative therapy including foscarnet or vidarabine may be of benefit [15–17].

A major limitation of our study was the retrospective design. Searching laboratory records rather than discharge codes might have identified more cases but was not practical at all sites. A surveillance program would be required to detect suspected in addition to proven cases [20]. The lack of HPeV testing at most sites precluded study of this virus. It is likely that other viral etiologies of meningitis or meningoencephalitis will eventually be identified. Methods of molecular testing varied by study center. Emerging molecular diagnostic panels may eventually improve diagnosis of CNS infections [21]. Infants with mild viral meningitis are not always recognized to have CNS infection. The total number of infants investigated at the 7 centers to yield the 174 with proven CNS infections is not known. The small sample size would have precluded detecting all differences in clinical presentation and outcome for EV versus HSV. Application of the International Encephalitis Consortium definition of encephalitis [22] to infants is problematic as it can be difficult to determine if they have altered level of consciousness or focal signs and they are less likely than older children to manifest fever or CSF pleocytosis. An EEG is not always performed. Therefore, we used a simplified definition for meningoencephalitis; this definition was highly dependent upon the decision to perform and the interpretation of head imaging (which was not always obtained) and recognition of seizures so could have missed or over-diagnosed cases. Rarely, aseptic meningitis can also result in seizures and head imaging abnormalities. Infants who had coagulopathy or were too systemically ill to have CSF obtained or who died before they had a diagnosis would have been missed. Molecular testing for HSV (and presumably for other viruses) can be falsely negative early in the course of infection. However, the inclusion of only proven cases was deemed to yield the most accurate data. There was inconsistent access to data on neurodevelopment follow-up and the timing and nature of this follow-up was not standardized between centers. Study results may not be applicable to resource poor settings.

## Conclusions

Proven viral CNS infections appear to be more common than proven bacterial infections in the first 90 days of life. Age < 21 days and presence of seizures or extra-CNS involvement are clues to HSV infection, even in the absence of CSF pleocytosis. However, not all infants with CNS HSV have seizures. Although most infants with EV CNS infections have good outcomes, the subset who have seizures and/or abnormal head imaging may have outcomes similar to those of infants with HSV meningoencephalitis and require neurodevelopmental follow-up. Further studies should address the contribution of HPeV to viral CNS infections and explore predictors of long-term morbidity.

## Data Availability

All data are stored in REDCap. An anonymized version is available from the corresponding author upon reasonable requests.

## Appendix

### ICD10CA codes used to identify potential cases

A170 Tuberculous meningitis

A203 Plague meningitis

A321 Listerial meningitis and meningoencephalitis

A390 Meningococcal meningitis

A870 Enteroviral meningitis

A871 Adenoviral meningitis

A872 Lymphocytic choriomeningitis

A878 Other viral meningitis

A879 Viral meningitis, unspecified

B003 Herpesviral meningitis

B010 Varicella meningitis

B021 Zoster meningitis

B051 Measles complicated by meningitis

B261 Mumps meningitis

B375 Candidal meningitis

B384 Coccidioidomycosis meningitis

G000 Haemophilus meningitis

G001 Pneumococcal meningitis

G002 Streptococcal meningitis

G003 Staphylococcal meningitis

G008 Other bacterial meningitis

G009 Bacterial meningitis, unspecified G00 Bacterial meningitis, not elsewhere classified

G01 Meningitis in bacterial diseases classified elsewhere

G020* Meningitis in viral diseases classified elsewhere

G021* Meningitis in mycoses

G028* Meningitis in other specified infectious and parasitic diseases classified elsewhere

G030 Nonpyogenic meningitis

G031 Chronic meningitis

G032 Benign recurrent meningitis [Mollaret]

G038 Meningitis due to other specified causes

G039 Meningitis, unspecified

A811 Subacute sclerosing panencephalitis

A830 Japanese encephalitis

A831 Western equine encephalitis

A832 Eastern equine encephalitis

A833 St Louis encephalitis

A834 Australian encephalitis

A835 California encephalitis

A838 Other mosquito-borne viral encephalitis

A839 Mosquito-borne viral encephalitis, unspecified

A840 Far Eastern tick-borne encephalitis [Russian spring-summer encephalitis]

A841 Central European tick-borne encephalitis

A848 Other tick-borne viral encephalitis

A849 Tick-borne viral encephalitis, unspecified

A850 Enteroviral encephalitis

A851 Adenoviral encephalitis

A852 Arthropod-borne viral encephalitis, unspecified

A858 Other specified viral encephalitis

A86 Unspecified viral encephalitis

A922 Venezuelan equine fever

A923 West Nile virus infection

B004 Herpesviral encephalitis

B011 Varicella encephalitis

B020 Zoster encephalitis

B050 Measles complicated by encephalitis

B262 Mumps encephalitis

B582 Toxoplasma meningoencephalitis

G040 Acute disseminated encephalitis

G042 Bacterial meningoencephalitis and meningomyelitis, not elsewhere classified

G048 Other encephalitis, myelitis and encephalomyelitis

G049 Encephalitis, myelitis and encephalomyelitis, unspecified

G050* Encephalitis, myelitis and encephalomyelitis in bacterial diseases classified elsewhere

G05.1* Encephalitis, myelitis and encephalomyelitis in viral diseases classified elsewhere

G052* Encephalitis, myelitis and encephalomyelitis in other infectious and parasitic diseases classified elsewhere

G058* Encephalitis, myelitis and encephalomyelitis in other diseases classified elsewhere

## Abbreviations

CNS: Central nervous system
CSF: Cerebrospinal fluid
EV: Enterovirus
HSV: Herpes simplex virus
IQR: Interquartile range
WBC: White blood cell count
